# Outage performance of UAV-NOMA networks over rician faded channel with hardware impairments, channel estimation error, and SIC imperfection

**DOI:** 10.1371/journal.pone.0348501

**Published:** 2026-05-08

**Authors:** Sk Thaherbasha, S.D. Nageena Parveen, Sivasubramanyam Medasani, Suman Turpati, Tathababu Addepalli, Manish Sharma, Sameena Pathan

**Affiliations:** 1 Department of Electronics and Communication Engineering, SR University, Warangal, Telangana, India; 2 Department of Electronics and Communication Engineering, SR University, Warangal, Telangana, India; 3 Department of CSE, K. S. School of Engineering and Management, Bengaluru, India; 4 Department of Electronics and Communication Engineering, Dr. K V Subba Reddy Institute of Technology, Kurnool, Andhra Pradesh, India; 5 Department of ECE, Aditya University, Surampalem, India; 6 Department of Electrical, Electronics and Communication Engineering, Galgotias University, Greater Noida, Uttar Pradesh, India; 7 Manipal Institute of Technology, Manipal Academy of Higher Education, Manipal, India; Northwestern Polytechnical University, CHINA

## Abstract

The escalating demand for enhanced coverage and high data rates in wireless networks is driving the adoption of advanced technologies like unmanned aerial vehicles (UAVs). Integrating UAVs with non-orthogonal multiple access (NOMA) has emerged as a promising solution to boost spectral efficiency and user connectivity. However, the practical performance of these UAV-assisted NOMA systems is critically constrained by real-world imperfections, including hardware impairments, inaccurate channel state information (CSI), and non-ideal successive interference cancellation (SIC). To address this, a reliable system design necessitates a precise outage probability analysis, which quantifies the impact of these impairments on both reliability and user experience. This work derives closed-form expressions for the outage probability of a multi-user UAV-assisted NOMA system operating over Rician fading channels, explicitly incorporating the effects of the aforementioned impairments. Analytical results are obtained for a two-user UAV-assisted NOMA system by considering the detrimental effect of hardware impairments along with imperfect CSI and SIC on system performance. These analytical results are further validated by simulated results.

## Introduction

**Conventional** terrestrial wireless networks are increasingly **demonstrating** their inability to satisfy the needs for lower latency, higher data speeds, and coverage throughout the globe. These **limitations** are especially pronounced in **densely populated urban environments**, rural regions, and **regions where the deployment of ground-based cellular infrastructure is economically prohibitive or physically impractical. In response to these challenges,** unmanned aerial vehicles (UAVs) have become a feasible solution since they may be used in a different of ways and can be enlarged to improve wireless coverage. In few areas where there isn’t sufficient ground-based infrastructure, UAVs may act as mobile base stations, allowing users to connect as necessitated. Also, since they are easy to move and set up, they may be changed to meet the requirements of users, geographical limits, and network problems that come up unnoticeably. Recent research has shown that UAV-supported communications need to be improved by optimizing trajectory design, power management, and resource allocation to make the network perform better and use less energy. The results show that hardware problems need to be fixed and operating processes need to be changed during crises. [1–3]. **Consequently, energy-efficient, intelligent, and robust wireless solutions are increasingly being adopted as the foundational design paradigm for UAV-enabled network architectures** [4–6].

**Non-orthogonal multiple access** (NOMA) **has emerged** as a pivotal technique for increasing the capacity of UAV-assisted networks. It works as an advanced multiple access method that serves several users at once on the same time-frequency resource, mostly using power-domain multiplexing. **Unlike conventional orthogonal multiple access** (OMA) systems, each user has their own time or frequency slots. **By superimposing multiple users’ signals at differentiated power levels and employing successive interference cancellation (SIC) at the receiver side, NOMA achieves substantially more efficient utilization of available spectral resources, translating directly into enhanced system capacity and improved spectrum efficiency** [7–11]. Using NOMA with UAVs may dramatically boost throughput and connectivity by letting more users connect with less latency. Table 1 summarizes the comparative summary of the principal modifications introduced to the UAV communication system that employs OMA/NOMA.

**Table 1:** Key developments of OMA/NOMA based UAV system.

Authors	Key focus	Parameters evaluated
[12]	UAV-swarm massive MIMO	Coverage, energy consumption, throughput
[13]	NOMA-UAV resource Optimization	Energy efficiency, spectral efficiency, fairness
[14]	Open-radio access network UAV deployment	Power allocation, latency, energy consumption
[15]	Multi carrier-NOMA UAV networks	Throughput, energy efficiency, fairness
[16]	Hardware-impaired NOMA-UAV	Outage probability, SINR degradation
[17]	UAV mobility in ultra dense-HetNets	Handover rate, delay, network stability
[18]	Orthogonal frequency division multiplexing-UAV IoT network	Energy harvesting, throughput
[19]	Index-modulation multi-UAV NOMA	Spectral efficiency, BER, coverage
[20]	Dual-NOMA UAV-IoT	Energy efficiency, power allocation
[21]	Spatial NOMA UAV	Power allocation, sum rate
[22]	Multi-way NOMA UAV	Outage probability with residual impairments
[23]	MIMO UAV-NOMA relay	Outage under Nakagami-m fading
[24]	Rate splitting multiple access-UAV positioning	Ergodic sum rate with antenna selection
[25]	Cognitive UAV-NOMA	Outage probability with interference

The performance of UAV-NOMA depends on the i) hardware noise, ii) the accuracy of channel state information (CSI), and iii) the effectiveness of **SIC** techniques. Hardware impairments include phase noise, I/Q imbalance, and amplifier nonlinearities due to the limitation of practical hardware design and imperfection in manufacturing. These lead to distortion noise, which degrades signal integrity and increases error rates. This is especially important in UAV-NOMA systems, since the lightweight and energy-constrained UAV hardware often relinquishes robustness for efficiency. The implication encompasses reduced SINR, higher power consumption, and lower data rates; thus, it is essential to account for hardware noise in system design for retaining the reliability of communication.

Imperfect CSI follows approximating biases that happen when a UAV’s location changes rapidly, which causes Doppler shifts and substantial interference from the ambient system. It is very important to have accurate CSI for NOMA systems since it helps with power allocation and interference management. No effective balance between fairness and throughput will be achieved in the face of performance deterioration, resulting in worse service quality for less capable system users. This is especially critical for UAV systems, as changing and unexpected circumstances make it even harder to keep correct CSI [26–29].

Imperfect SIC is caused by residual interference that may remain owing to incorrect cancellation processes. This normally results from errors in decoding, accumulation of noise, or limited computation capabilities of UAVs. In fact, SIC is important in NOMA systems for the realization of effective spectrum reuse and fairness maintenance toward weaker users. The imperfection in SIC deteriorates the qualities of signals for users in poor channel conditions, thus reducing the efficiency of the system in its totality. In UAV-based systems, the fast-changing topology and limited on-board processing further worsen these challenges, making SIC accuracy improvement of paramount importance [30–34].

Therefore, precise calculations in the performance metrics are very important for UAV-assisted NOMA systems under realistic conditions. The outage probability is one of the important performance metrics, which indicates the probability that a user’s signal falls below the required threshold to **maintain a service**. The outage probability depends on various factors, such as hardware impairments, CSI accuracy, and SIC effectiveness. In this work, we derive outage probability expressions in closed-form for a UAV-assisted NOMA system that accounts for hardware impairments’ impact, imperfect CSI, as well as imperfect SIC over Rician fading channels.

**In the existing literature, most of the reported works analyse the impact of system impairments such as imperfect CSI, imperfect SIC, and hardware impairments individually in UAV-NOMA systems. In many cases, the outage probability analysis is performed by considering only one or two impairments at a time while assuming ideal conditions for the remaining system components. Moreover, a significant portion of the existing studies primarily focus on two-user NOMA systems, which simplifies the analysis but does not fully represent practical multi-user communication scenarios. However, in practical wireless communication systems, particularly in UAV-assisted networks, these impairments occur simultaneously and collectively affect the overall system performance. In this work, we consider it a more realistic system model by incorporating UAV hardware impairments, imperfect CSI, and imperfect SIC simultaneously in the analysis of a multi-user UAV-assisted NOMA system. To accurately capture the effect of these impairments, we have formulated the signal to interference plus noise ratio (SINR) expressions by incorporating the impact of all impairments together. This formulation enables a more precise representation of the received signal conditions in practical UAV communication environments. Furthermore, we derive closed-form analytical expressions for the outage probability over a Rician faded channel, which is suitable for multi-user UAV-ground communication scenarios.**

The obtained results provide valuable insight into the impact of parameters and various system-related impairments on performance in outage conditions and can be very helpful in fully characterizing the system from works [26–34]. In this work, we consider Rician faded channel which accurately model real-world communication scenarios and it have a strong **line-of-sight (LoS)** path along with scattered multipath components.

This research demonstrates a thorough analytical foundation UAV-assisted NOMA system for a dual-user. We give closed-form equations for the outage likelihood that evidently show how hardware problems, imperfect **SIC**, and lack of **CSI** may all make things adverse. Monte Carlo simulations thoroughly analyse the accuracy of the **proposed** model. **These results** illustrate how these difficulties in the actual world make systems less reliable. This is critical information for constructing strong UAV-NOMA networks. This paper puts forward targeted recommendations for improving several critical system components, including channel estimation accuracy, the appropriate selection of power allocation coefficients, and the **practical implementation** of SIC at the receiver.

### System Model

This Section (System model) includes the mathematical modelling for the power-domain NOMA multi-user UAV communication. This setup involves a single UAV acting as the transmitter, providing downlink communication to *K* users positioned at different distances, $d_{k}$, from the UAV. The distance impacts the received signal quality due to path loss and channel fading. The power allocation to each user is determined based on the channel conditions as users closer to the UAV (stronger channel conditions) are assigned less transmission power, while users farther away (weaker channel conditions) are allocated more power to ensure fairness and connectivity. Assume the UAV flies in a circular trajectory at a fixed altitude *H*, with a radius *r*_*1*_, while *K* users are randomly distributed within a circular ground area of radius *r*_*2*_. The UAV’s position in 3D Cartesian coordinates is given as $\left(x_{u},y_{u},z_{u}\right)=\left(r_{\text{1}}cos\theta_{u},r_{\text{1}}sin\theta_{u},H\right)$, where $\theta_{u}$ represents the azimuth angle of the UAV’s position along its trajectory. For each *k*-user, where *k*∈*{1,2,…,K},* the position is represented by $\left(x_{k},y_{k},z_{k}\right)=\left(r_{\text{2}}cos\;\theta_{k},r_{\text{2}}sin\;\theta_{k},H\right)$. Using this, the Euclidean distance between the UAV and the *k*-th user is (Equation (1))


$$\begin{array}{c} d_{k}=\sqrt{(x_{u}-x_{k}{)}^{\text{2}}+(y_{u}-y_{k}{)}^{\text{2}}+(z_{u}-z_{k}{)}^{\text{2}}}\\ \text{\textbackslash{}hspace\{0.17em}\;\;\;\;\;\}=\sqrt{(r_{\text{1}}cos\;\theta_{u}-r_{\text{2}}cos\;\theta_{k}{)}^{\text{2}}+(r_{\text{1}}sin\;\theta_{u}-r_{\text{2}}sin\;\theta_{k}{)}^{\text{2}}+H^{\text{2}}}\text{\textbackslash{}hspace\{0.17em}\;\}. \end{array}$$ (1)

The transmitted signal in this system is modelled as a superposition of signals intended for all users, weighted by their respective power allocation coefficients, $\beta_{k}$, such that $\beta_{\text{1}}>\beta_{\text{2}}.....>\beta_{K}$, and $\begin{array}{c} \sum \overset{K}{\underset{k=1}{\mathrm{\backslash nolimits}}}\beta_{k}=1 \end{array}$. The UAV-assisted NOMA based system’s transmitted signal can be represented by Equation (2) as:


$$S=\sum_{k=1}^{K}\sqrt{\beta_{k}P}s_{k},$$ (2)

where $s_{k}$ is the message signal for the *k*-th user. In a UAV-assisted NOMA system, the received signal at the *k*-th user is impacted by both imperfect CSI and SIC. Imperfect CSI introduces additional interference, affecting the accuracy of SIC and hindering precise signal recovery. The CSI error, denoted by ξ, is typically modelled as a complex Gaussian distribution, characterized by zero mean and variance, $CN\left(0,\text{\textbackslash{}hspace\{0.17em}\}\overset{\text{2}}{\underset{e}{\sigma }}\right)$ it follows $h_{k}={\hat{h}}_{k}+\xi$. This error reflects the deviation between the estimated and actual channel conditions. Imperfect SIC arises due to the inability to fully eliminate interference from previously decoded signals. The residual interference is treated as an additional noise component, modelled as a Gaussian distribution with zero mean and variance, $\overset{\text{2}}{\underset{k}{\zeta }}$. This residual noise depends on the quality of the SIC process and the system’s hardware impairments.

**Fig 1:**
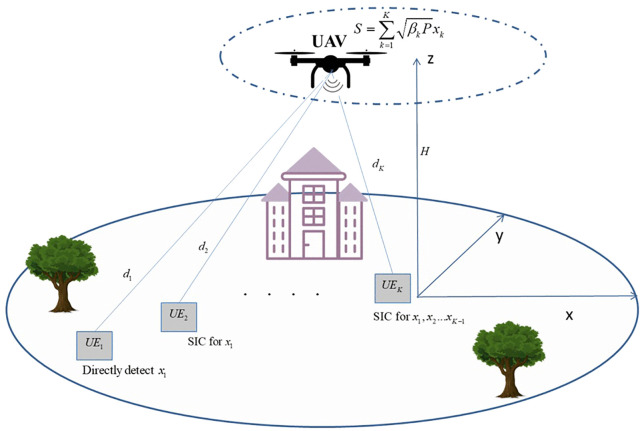
UAV-NOMA based communication system with hardware impairments.

Fig 1 illustrates UAV-NOMA model where UAV-assisted NOMA systems, the communication channel between the UAV and users is subject to various impairments that can distort the transmitted signal. The standard communication model assumes an ideal system where the received signal is given by R=h\hspace{0.17em}S+n, where *h* represents the channel gain, and *η* is additive noise. However, in real-world systems, hardware imperfections such as I/Q imbalance, phase noise, and non-linearity’s in the power amplifier can distort both the transmitted and received signals. In this work, these impairments are modelled as distortion noise at both the transmitter and receiver. Specifically, the distortion noise at the transmitter ($\tau_{t}$) is represented by $CN\left(0,\text{\textbackslash{}hspace\{0.17em}\}\overset{\text{2}}{\underset{t}{\eta }}P\right)$, and at the receiver ($\tau_{r}$) by $CN\left(0,\text{\textbackslash{}hspace\{0.17em}\}\overset{\text{2}}{\underset{r}{\eta }}P\right)$, where $\overset{\text{2}}{\underset{t}{\eta }}$ and $\overset{\text{2}}{\underset{r}{\eta }}$ characterize the levels of distortion at the transmitter and receiver, respectively. The combined distortion noise is correlated with the channel gain *h*, making it different from the classical channel model, where noise is independent of the channel. This results in a more complex noise structure that must be accounted for in the system’s performance analysis. The total distortion is given by $E\left[{\left|\tau_{t}+\tau_{r}\right|}^{\text{2}}\right]=P{\left|h\right|}^{\text{2}}(\overset{\text{2}}{\underset{t}{\eta }}+\overset{\text{2}}{\underset{r}{\eta }})$ which highlights the dependence of distortion on both the transmitted signal power and the channel gain. This can be simplified into an equivalent model R=h\hspace{0.17em}(S+τ)+η where $\tau \;CN\left(0,\text{\textbackslash{}hspace\{0.17em}\}\eta^{\text{2}}P\right)$ and $\eta =\sqrt{\overset{\text{2}}{\underset{t}{\eta }}+\overset{\text{2}}{\underset{r}{\eta }}}$ [35,36].

At the receiver, the *k*-th user processes a composite signal that includes the desired signal, inter-user interference, hardware-induced distortions, additive white Gaussian noise (AWGN), CSI errors, and SIC residual interference. With these considerations, mathematically, the received signal can be expressed by Equation (3) and is given below


$$R_{k}=({\hat{h}}_{k}\overset{-\alpha /2}{\underset{k}{d}}+\xi )\sum_{k=1}^{K}\left(\sqrt{\beta_{k}P}s_{k}+n_{k}\right)+n_{k}\text{\textbackslash{}hspace\{0.17em}\}.$$ (3)

To decode signals, users employ SIC. The farthest user does not perform SIC, while other users decode and subtract signals from weaker users before decoding their own signal. Imperfect SIC introduces residual interference, modelled as an additional noise term, which impacts SINR.

The SINR for the *k*-th user in a UAV-assisted NOMA system calculated from Equation (4) quantifies the signal quality at the receiver while considering the interference from other users sharing the channel and the background noise. In this setup, the SINR for the *k*-th user can be **derived as** [37–39].


$$\begin{array}{c} \gamma_{j\to k}=\frac{\text{Required\textbackslash{}hspace\{0.17em}signal}{\}}\text{Noise+\textbackslash{}hspace\{0.17em}Interference\;due\;to\;error\;in\;CSI\;and\;SIC\;+\;Hardware\;noise\;\}\text{\textbackslash{}hspace\{0.17em}\},\\ \text{\textbackslash{}hspace\{0.17em}\;\;\;\;\;\;\;\;\;\;\}=\frac{\beta_{k}{\left|{\hat{h}}_{k}\right|}^{\text{2}}\overset{-\alpha /2}{\underset{k}{d}}\rho }{1+{\left|{\hat{h}}_{k}\right|}^{\text{2}}\overset{-\alpha /2}{\underset{k}{d}}\rho \sum_{i=k+1}^{K}\beta_{i}+\overset{\text{2}}{\underset{e}{\sigma }}\rho +\kappa^{\text{2}}\rho \overset{-\alpha /2}{\underset{k}{d}}\left(\overset{\text{2}}{\underset{e}{\sigma }}+{\left|{\hat{h}}_{k}\right|}^{\text{2}}\right)+{\left|{\hat{h}}_{k}\right|}^{\text{2}}\overset{-\alpha /2}{\underset{k}{d}}\rho \sum_{l=1}^{j-1}\beta_{l}{\left|\xi_{l}\right|}^{\text{2}}}\text{\textbackslash{}hspace\{0.17em}\;\;\;\}, \end{array}$$ (4)

where **signal to noise ratio (SNR)**, $\rho =\frac{p}{\overset{\text{2}}{\underset{k}{\sigma }}}$. The channel is modelled to include large-scale path loss and small-scale fading, often characterized by Rician distribution. These fading effects, combined with UAV mobility, make the channel conditions dynamic and challenging. This mathematical framework provides the foundation for analysing outage probability and optimizing UAV-NOMA system design.

### Channel modelling

This Section (Channel modelling) discusses both large-scale route loss and small-scale fading effects which can affect the wireless communication networks between UAVs and ground users in UAV-based NOMA systems. The Rician fading model is commonly used to describe UAV-to-ground connections because of the predominance of the **LoS** component. Both random multipath effects due to scatterers at the receiver and a deterministic LoS component are taken into consideration by the Rician model. The probability density function (PDF) under Rician fading of the squared channel gain, ${\hat{h}}_{k}$, can be illustrated as Equation (5):


$$f_{{\left|{\hat{h}}_{k}\right|}^{\text{2}}}\left(x\right)=\frac{1+\kappa }{{\overset{―}{\lambda }}_{k}}e^{-\kappa }e^{-\left(\frac{1+\kappa }{{\overset{―}{\lambda }}_{k}}\right)x}I_{\text{0}}\left(2\sqrt{\frac{\kappa (1+\kappa )}{{\overset{―}{\lambda }}_{k}}x}\right),$$ (5)

where *K* signifies the Rician factor, ${\overset{―}{\lambda }}_{k}$ is the *k*-th user average power and *I0* is the modified Bessel function of the zero order first kind. The cumulative distribution function (CDF) under Rician fading of the squared channel gain is defined by Equation (6)


$$F_{{\left|{\hat{h}}_{k}\right|}^{\text{2}}}\left(x\right)=1-Q\left(\sqrt{2\kappa },\sqrt{2\frac{1+\kappa }{{\overset{―}{\lambda }}_{k}}x}\right),$$ (6)

where Q(a,b) is the Marcum Q-function in first order. The Marcum Q-function can be approximated and manipulated using properties from [31] and [36], to derive an expanded form of the CDF given in Equation (7). The generated expression for the CDF is:


$$F_{{\left|{\hat{h}}_{k}\right|}^{\text{2}}}\left(x\right)=1-e^{-\kappa }e^{-\left(\frac{1+\kappa }{{\overset{―}{\lambda }}_{k}}\right)x}\sum_{k=0}^{\infty }\sum_{q=0}^{\infty }\frac{\kappa^{k+q}{\left(\frac{1+\kappa }{{\overset{―}{\lambda }}_{k}}\right)}^{q}x^{q}}{q!\Gamma (k+q+1)}\text{\textbackslash{}hspace\{0.17em}\}.$$ (7)

This expression captures the mixed effect of the deterministic LoS path and the stochastic multipath elements in UAV-to-ground communication.

### Outage Probability Analysis

This Section which is outage probability analysis discusses the equation model analysis of a UAV-assisted NOMA. In the context of a UAV-assisted NOMA multi-user system, one of the significant metrics toward the analysis of the system for its reliability and robustness is the outage probability. It offers the probability that a user lacks to achieve the minimum required SINR for effective communication. This metric is notably crucial in UAV communication networks, where dynamic channel conditions and interference management are essential in supporting multiple users successively. The outage probability is defined as, the probability that the instantaneous SINR declines below a predefined threshold for the *k*-th user $\overset{k}{\underset{th}{\gamma }}$ which is the minimum SINR needed for acceptable performance. Mathematically, the outage probability for the *k*-th user can be expressed as Equation (8):


$$\overset{k}{\underset{out}{P}}=\text{Pr}\left(\gamma_{1\to k}\leq \overset{\text{1}}{\underset{th}{\gamma }}\cap \gamma_{2\to k}\leq \overset{\text{2}}{\underset{th}{\gamma }}\ldots \cap \gamma_{k}\leq \overset{m}{\underset{th}{\gamma }}\right)\text{\textbackslash{}hspace\{0.17em}\},$$ (8)

where $\overset{k}{\underset{th}{\gamma }}$ is the threshold value of the SINR for the *k*-th user. Using (4), the event $\gamma_{j\to k}\geq \overset{k}{\underset{th}{\gamma }}$ is replaced as


$$\gamma_{j\to k}>\overset{k}{\underset{th}{\gamma }}\text{\textbackslash{}hspace\{0.17em}\},$$

$$\frac{\beta_{k}{\left|{\hat{h}}_{k}\right|}^{\text{2}}\overset{-\alpha /2}{\underset{k}{d}}\rho }{1+{\left|{\hat{h}}_{k}\right|}^{\text{2}}\overset{-\alpha /2}{\underset{k}{d}}\rho \sum_{i=k+1}^{K}\beta_{i}+\overset{\text{2}}{\underset{e}{\sigma }}\rho +\kappa^{\text{2}}\rho \overset{-\alpha /2}{\underset{k}{d}}\left(\overset{\text{2}}{\underset{e}{\sigma }}+{\left|{\hat{h}}_{k}\right|}^{\text{2}}\right)+{\left|{\hat{h}}_{k}\right|}^{\text{2}}\overset{-\alpha /2}{\underset{k}{d}}\rho \sum_{l=1}^{j-1}\beta_{l}{\left|\xi_{l}\right|}^{\text{2}}}>\overset{k}{\underset{th}{\gamma }}\text{\textbackslash{}hspace\{0.17em}\},$$

$${\left|{\hat{h}}_{k}\right|}^{\text{2}}>\frac{\overset{k}{\underset{th}{\gamma }}\left(1+\rho \overset{\text{2}}{\underset{e}{\sigma }}\left(1+\eta^{\text{2}}\rho \overset{-\alpha /2}{\underset{k}{d}}\right)\right)}{\beta_{k}\overset{-\alpha /2}{\underset{k}{d}}\rho -\overset{k}{\underset{th}{\gamma }}\overset{-\alpha /2}{\underset{k}{d}}\rho \left({\tilde{\beta }}_{j}+\Lambda +\eta^{\text{2}}\right)}\text{\textbackslash{}hspace\{0.17em}\},$$ (9)

_where_


$${\tilde{\beta }}_{j}=\sum_{j=k+1}^{K}\beta_{j}\text{\textbackslash{}hspace\{0.17em}\},$$ (10)

$$\Lambda =\sum_{l=1}^{j-1}\beta_{l}{\left|\xi_{l}\right|}^{\text{2}}\text{\textbackslash{}hspace\{0.17em}\}.$$ (11)

Using Equation (8), Equation (9), Equation (10) and Equation (11) the outage probability is expressed as Equation (12).


$$\overset{k}{\underset{out}{P}}=1-Pr\left(\underset{j=1,2..K}{⋂}{\left|{\hat{h}}_{k}\right|}^{\text{2}}>\frac{\overset{k}{\underset{th}{\gamma }}\left(1+\rho \overset{\text{2}}{\underset{e}{\sigma }}\left(1+\eta^{\text{2}}\rho \overset{-\alpha /2}{\underset{k}{d}}\right)+\rho \sum_{l=1}^{j-1}\beta_{l}{\left|\xi_{l}\right|}^{\text{2}}\right)}{\beta_{k}\overset{-\alpha /2}{\underset{k}{d}}\rho -\overset{k}{\underset{th}{\gamma }}\overset{-\alpha /2}{\underset{k}{d}}\rho \left({\tilde{\beta }}_{j}+\Lambda +\eta^{\text{2}}\right)}\right)\text{\textbackslash{}hspace\{0.17em}\}.$$ (12)

By implementing the SIC technique in the NOMA-based UAV communication system, efficient multi-user communications are realized owing to mitigated signals from interference. For the *k*-th user, the SIC performs signal decoding and cancellation first from the users whose channel condition is finer than its own, then decodes its signal. It thus requires ordering of users in decreasing order of their channel strengths and decoding the signal of users with better channel conditions first. The corresponding SINR of these stronger users has to be larger than a pre-defined minimum threshold to ensure the success of SIC. Otherwise, the SIC procedure may not work, and decoding errors may propagate due to interference. With this, it must satisfy: $\beta_{k}\overset{-\alpha /2}{\underset{k}{d}}\rho >\overset{k}{\underset{th}{\gamma }}\overset{-\alpha /2}{\underset{k}{d}}\rho \left({\tilde{\beta }}_{j}+\Lambda +\eta^{\text{2}}\right)$ and it follows.


$${\left|{\hat{h}}_{k}\right|}^{\text{2}}>\Omega_{k}\phi \text{\textbackslash{}hspace\{0.17em}\},$$ (13)

where


$$\Omega_{k}=1+\rho \overset{\text{2}}{\underset{e}{\sigma }}\left(1+\eta^{\text{2}}\rho \overset{-\alpha /2}{\underset{k}{d}}\right)\text{\textbackslash{}hspace\{0.17em}\},$$

$$\phi =\underset{j}{max}\frac{\overset{k}{\underset{th}{\gamma }}}{\beta_{k}\overset{-\alpha /2}{\underset{k}{d}}\rho -\overset{k}{\underset{th}{\gamma }}\overset{-\alpha /2}{\underset{k}{d}}\rho \left({\tilde{\beta }}_{j}+\Lambda +\eta^{\text{2}}\right)}\text{\textbackslash{}hspace\{0.17em}\}.$$ (14)

Using Equation (12), Equation (13) and Equation (14) the outage probability is expressed as Equation (15).


$$\overset{k}{\underset{out}{P}}=F_{h_{k}}\left(\Omega_{k}\phi \right)\text{\textbackslash{}hspace\{0.17em}\}.$$ (15)

In the UAV-based NOMA system, the channels are ordered in descending order of their quality. The strongest channel is placed first, while the weakest channel is positioned last. This ordering will facilitate the analysis and modelling of the outage probability by leveraging the **CDF** of these ranked channels. With ordered channel gains, the outage performance for each user can be well investigated to ensure that the system is sufficiently reliable and capable of decoding signals in UAV-assisted communication scenarios **as.**


$${\overset{―}{F}}_{{\hat{h}}_{k}}\left(x\right)\text{\textbackslash{}hspace\{0.17em}\}=\text{\textbackslash{}hspace\{0.17em}\}\frac{K!}{(K-k)!(k-1)!}\sum_{l=0}^{K-k}\left(\begin{array}{c} @c@K-k\\ l \end{array}\right)\text{\textbackslash{}hspace\{0.17em}\frac{(-1{)}^{l}}{k+l}{\left[F_{{\hat{h}}_{k}}\left(x\right)\right]}^{k+l}\}\text{\textbackslash{}hspace\{0.17em}\}.$$ (16)

**Using Equation (15) and equation (16),** the outage probability expression is written as Equation (17).


$$\overset{k}{\underset{out}{P}}=\text{\textbackslash{}hspace\{0.17em}\}\frac{K!}{(K-k)!(k-1)!}\sum_{l=0}^{K-k}\left(\begin{array}{c} @c@K-k\\ l \end{array}\right)\text{\textbackslash{}hspace\{0.17em}\frac{(-1{)}^{l}}{k+l}{\left[F_{{\hat{h}}_{k}}\left(\Delta_{k}\phi \right)\right]}^{k+l}\}\text{\textbackslash{}hspace\{0.17em}\}.$$ (17)

Using **(17)**, the final expression for the outage probability of UAV-assisted NOMA system is obtained as Equation (18).


$$\overset{k}{\underset{out}{P}}=\text{\textbackslash{}hspace\{0.17em}\}\frac{K!}{(K-k)!(k-1)!}\sum_{l=0}^{K-k}\left(\begin{array}{c} @c@K-k\\ l \end{array}\right)\text{\textbackslash{}hspace\{0.17em}\frac{(-1{)}^{l}}{k+l}{\left[1-e^{-\kappa }e^{-\left(\frac{1+\kappa }{{\overset{―}{\lambda }}_{k}}\right)\Omega_{k}\phi }\sum_{k=0}^{\infty }\sum_{q=0}^{\infty }\frac{\kappa^{k+q}{\left(\frac{1+\kappa }{{\overset{―}{\lambda }}_{k}}\right)}^{q}(\Omega_{k}\phi {)}^{q}}{q!\Gamma (k+q+1)}\right]}^{k+l}\}\text{\textbackslash{}hspace\{0.17em}\}.$$ (18)

## Results and discussions

In this Section, results for the UAV-assisted NOMA system are obtained over Rician fading channel. The near user, positioned closer to the UAV, experiences lower path loss and thus receives a smaller power allocation, while the far user, located farther away, is allocated more power to overcome increased path loss. Simulations conducted in MATLAB, incorporating UAV hardware impairments, imperfect CSI and SIC. Outage probability results were calculated for two user NOMA analytically and verified through simulations across different SNR values, providing a comprehensive comparison. Outage probability was assessed under four distinct conditions: (a) Perfect CSI and SIC, (b) Imperfect CSI, (c) Imperfect SIC, and (d) UAV to User distance, where increased distance significantly degraded SINR for both users due to heightened path loss, underscoring the importance of robust CSI and SIC strategies to maintain reliable connectivity in UAV-assisted NOMA systems. The simulated parameters used in this UAV-assisted NOMA system analysis are presented in Table 2.

**Table 2:** Simulation parameters for two user UAV-assisted NOMA based system.

Parameter	Value
SNR, 𝜌	0 to 30dB
**Far user, power allocation,** $\beta_{\text{1}}$	0.8
**Near user, power allocation,** $\beta_{\text{2}}$	0.2
Error in CSI,	10^−6^–10^−1^
Error in SIC,	10^−6^–10^−1^
Hardware impairment at UAV, *κ*	0.01
Distance of the Near user, *d*_*2*_	100 meters
Distance of the Far user, *d*_*1*_	400 meters
Vertical height of the UAV from ground	300 meters
**Circular trajectory radius of UAV,** $r_{\text{1}}$	**100 meters**
**Radius of user distribution** $r_{\text{2}}$	**500 meters**

**Fig 2:**
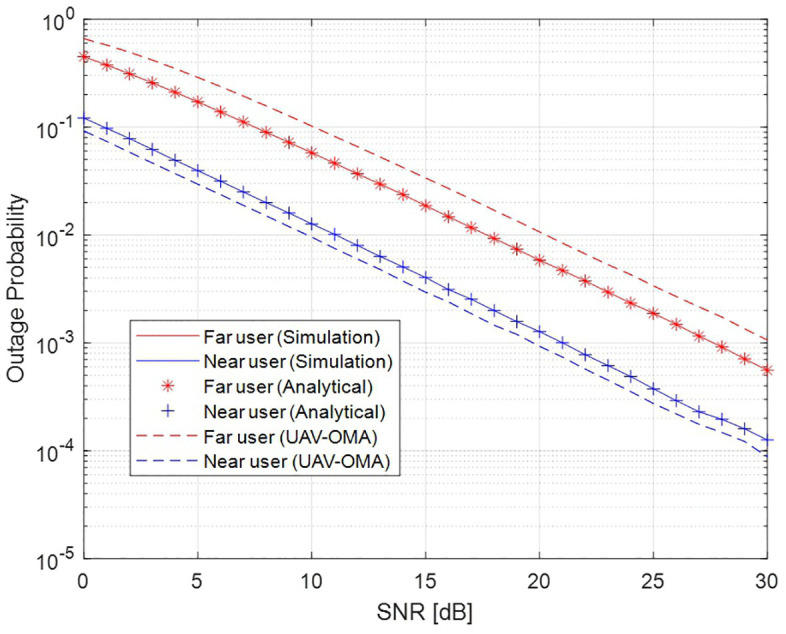
Outage probability of two user UAV-based NOMA system.

Fig 2 gives the outage probability of the proposed two-user UAV-based NOMA system with **SNR, and perfect CSI and SIC.** As the SNR increases, the outage probabilities of both users decline significantly, reflecting the direct relationship between signal strength and link reliability. However, for larger values of SNR, the outage probability decreases drastically and, in fact, signifies an improvement in the context of reliability in communication. Normally, the far user suffers from higher outage probability as compared to the near user even with higher power allocation due to larger path loss. **The outage probability of UAV-based NOMA system is also compared with the conventional UAV-OMA scheme. Here, UAV-NOMA system performs better than the UAV-OMA system for both users which is mainly due to the power-domain multiplexing capability of NOMA. In contrast, the OMA system allocates orthogonal resources to each user, which reduces the effective transmission rate and leads to higher outage probability.**

**Fig 3:**
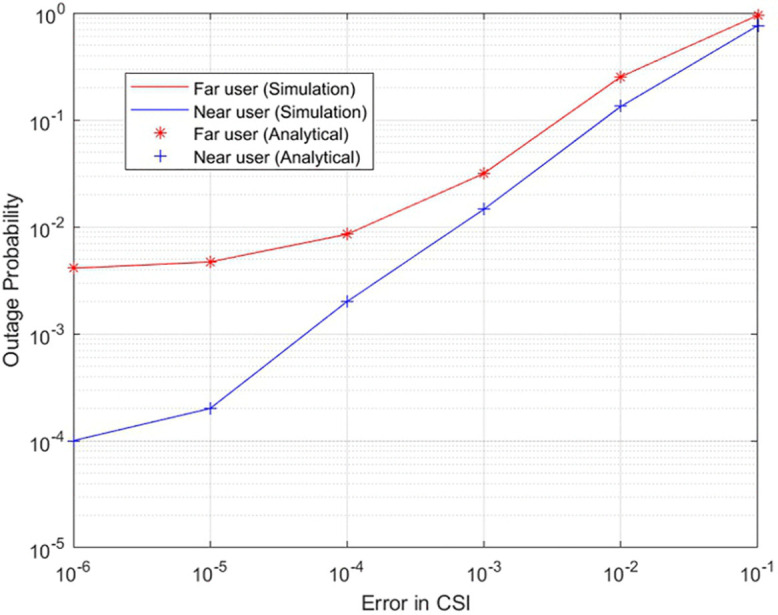
Outage probability of two user UAV-based NOMA system with imperfect-CSI.

Fig 3 **illustrates** the outage probability of a two-user UAV-based NOMA system with imperfect CSI. Here, the outage probability for both the near and far users is shown as a function of SNR, which ranges from 0 to 30dB. Unlike in the perfect CSI case, inaccuracy in channel estimation brings higher outage probability across all SNR values. While in the lower SNR levels, the outage probability is much larger for both users, this clearly reflects the challenge in communication when the signal quality is bad. By increasing the SNR, there is a remarkable decline in the outage probability. However, the decline observed here is not as high as in the case of perfect CSI. It therefore follows that while an increased SNR boosts the system performance, imperfect channel information bounds the optimum power allocation and interference management. Far users remain higher in outage probability due to easy susceptibility to path loss and interference.

**Fig 4:**
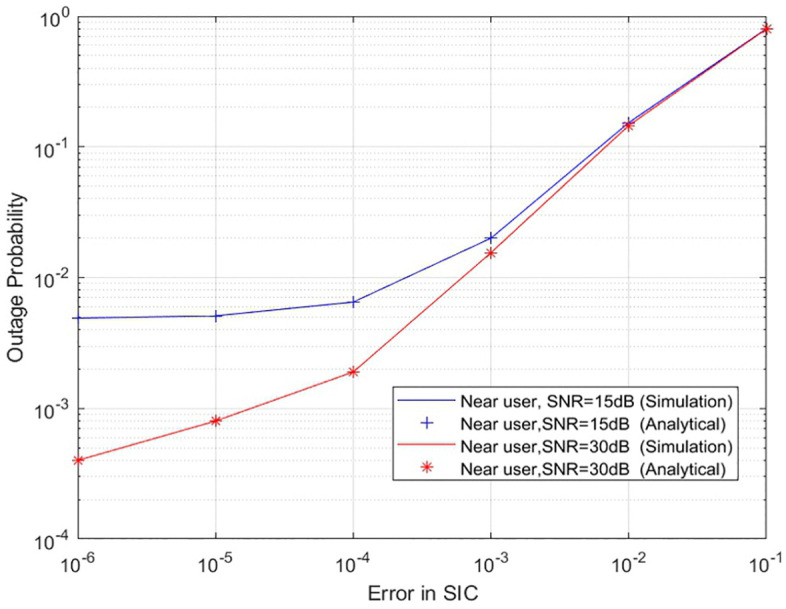
Outage probability of two user UAV-based NOMA system with imperfect-SIC.

Fig 4 shows the outage probability of a two-user UAV-based NOMA system when the SIC is imperfect. The outage probability for both users is extremely high at low SNR due to the small power in the signal and inability to effectively cancel interference from the near user’s signal. As SNR increases, **a gradual reduction** in outage probability **is observed**, **however, this improvement is notably less pronounced compared to the perfect SIC case.** This behaviour can be attributed to the persistent residual interference introduced by imperfect cancellation, which places a floor on the achievable reliability, particularly for the far user. **Consequently, even at higher SNR levels, the far user’s outage performance remains significantly degraded relative to the near user, underscoring the sensitivity of NOMA systems to SIC imperfections and the importance of accurate interference cancellation in practical UAV-assisted deployments.**

**Fig 5:**
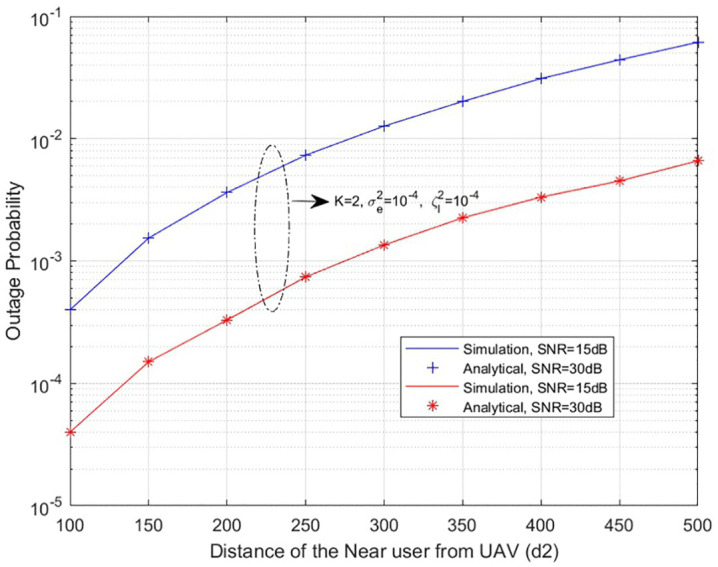
Outage probability of near user with respect to distance from UAV.

**Fig 6:**
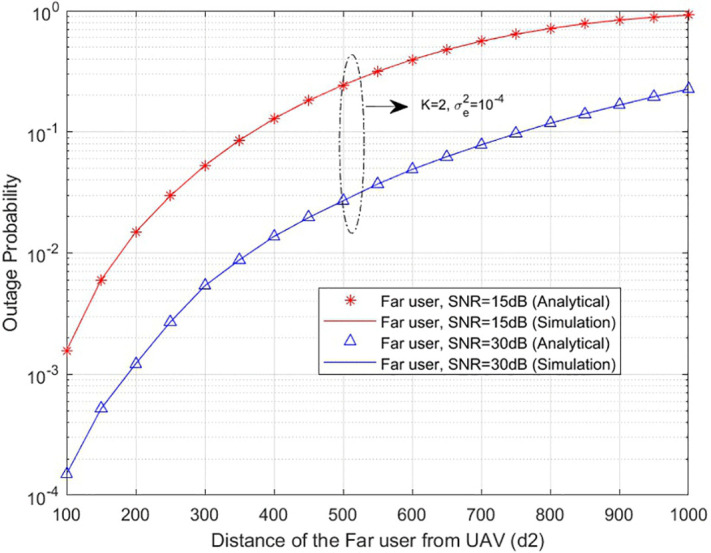
Outage probability of far user with respect to distance from UAV.

**The outage probability of both near and far users is evaluated by varying the distance between the UAV and the users, which reflects the effect of UAV positioning and mobility on the communication link. This analysis is presented in Fig 5 and Fig 6** **, where the outage probability is analysed with respect to different user distances from the UAV.**

Fig 5 illustrates the outage probability of the near user versus the **distance from the UAV**. With the increase in the distance between the near user and the UAV, the outage probability tends to increase. This trend is attributed to increasing path loss associated with the increased separation, which tends to diminish the quality of the signal received by the user. With a lower valued SNR, the associated outage probability is much larger, showing that the contribution from a user at some location has difficulty maintaining his link due to poor signal strength, and an outage occurs because the receiver fails to perform reliably. **As the SNR increases, a corresponding reduction in outage probability is observed across all separation distances. However, the performance gain diminishes progressively with increasing UAV-to-user separation, indicating that beyond a certain distance threshold, SNR improvements alone are insufficient to fully compensate for the degradation induced by path loss.**

The outage probability of the far user with respect to the distance from the UAV is given by Fig 6. **Across all distances considered, the far user consistently exhibits a higher outage probability than the near user, a direct consequence of the greater path loss experienced over longer transmission distances, which degrades the received signal quality. This disparity is particularly evident at low SNR levels, where the combined effect of distance and weak signal power renders it increasingly difficult to maintain a reliable communication link.**

**Fig 7:**
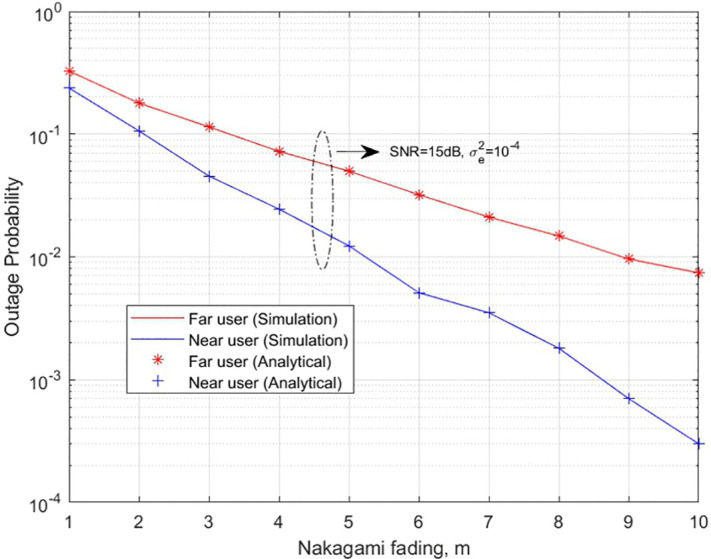
UAV-based NOMA system Outage probability of two user over Nakagami-*m* faded channel.

In Fig 7, outage probability of the two-user UAV-based NOMA system over Nakagami-*m* faded channel, obtained from the results on Rician fading channel by $1+\kappa =m+\sqrt{m^{\text{2}}-m}$. **Here**, outage probabilities for the near and far users **are obtained** with SNR levels from 0 to 30dB, affected by Nakagami-*m* fading. This analysis has elucidated the impact of Nakagami-*m* fading, which models the variation in signal strength arising from the multipath propagation of signals. The complete analysis of the two-user UAV assisted NOMA system is illustrated in the Table 3.

**Table 3:** Outage analysis of two-user UAV assisted NOMA system with various impairments.

Parameter	Outcome
Perfect CSI and SIC	Significant reduction in outage probability as SNR increases; far user has higher outage probability due to distance.
Imperfect CSI	Increased outage probabilities, particularly at lower SNRs; far user more affected by path loss and interference.
Imperfect SIC	Higher outage probabilities due to residual interference; far user shows elevated outage rates across all SNRs.
Near User Distance from UAV	Outage probability increases with distance; proximity to the UAV is crucial for reliable communication.
Far User Distance from UAV	Consistently higher outage probability compared to the near user; greater distance leads to more significant challenges.
Nakagami-*m* Fading	Increased outage probabilities compared to ideal conditions; emphasizes the need for strategies to manage fading effects even with improved SNR.

## Conclusion

In this Section discusses the conclusions of the proposed work where the research facilitates a comprehensive analysis of the UAV-assisted NOMA systems outage performance under practical impairments, comprising hardware limitations, imperfect SIC, and imperfect CSI, over Rician fading channels. By getting closed-form formulations for outage probability, the work evaluates the effects of these impairments, offering significant insights into the reliability and construct of such systems. The analytical results are validated through simulations, **with hardware limitations**, and residual interference considerably impact system reliability. This **analysis** emphasize the importance of robust methodologies for accurate CSI estimation, power allocation, and effective SIC implementation to elevated communication reliability. This research contributes to the development of more reliable and effective UAV-NOMA systems, by accounting for realistic conditions and employing Rician fading channels to model **LoS** and multipath environments. These evaluations are crucial for adopting robust communication infrastructures in dynamic and constrained conditions, allowing improved connectivity, enhanced spectral efficiency, and better user experience.
